# Adenosine-mediated modulation of ventral horn interneurons and spinal motoneurons in neonatal mice

**DOI:** 10.1152/jn.00574.2014

**Published:** 2015-08-26

**Authors:** Emily C. Witts, Filipe Nascimento, Gareth B. Miles

**Affiliations:** School of Psychology and Neuroscience, University of St Andrews, St Andrews, Fife, United Kingdom

**Keywords:** spinal cord, motor control, neuromodulation, purines

## Abstract

Neuromodulation allows neural networks to adapt to varying environmental and biomechanical demands. Purinergic signaling is known to be an important modulatory system in many parts of the CNS, including motor control circuitry. We have recently shown that adenosine modulates the output of mammalian spinal locomotor control circuitry (Witts EC, Panetta KM, Miles GB. *J Neurophysiol* 107: 1925–1934, 2012). Here we investigated the cellular mechanisms underlying this adenosine-mediated modulation. Whole cell patch-clamp recordings were performed on ventral horn interneurons and motoneurons within in vitro mouse spinal cord slice preparations. We found that adenosine hyperpolarized interneurons and reduced the frequency and amplitude of synaptic inputs to interneurons. Both effects were blocked by the A_1_-type adenosine receptor antagonist DPCPX. Analysis of miniature postsynaptic currents recorded from interneurons revealed that adenosine reduced their frequency but not amplitude, suggesting that adenosine acts on presynaptic receptors to modulate synaptic transmission. In contrast to interneurons, recordings from motoneurons revealed an adenosine-mediated depolarization. The frequency and amplitude of synaptic inputs to motoneurons were again reduced by adenosine, but we saw no effect on miniature postsynaptic currents. Again these effects on motoneurons were blocked by DPCPX. Taken together, these results demonstrate differential effects of adenosine, acting via A_1_ receptors, in the mouse spinal cord. Adenosine has a general inhibitory action on ventral horn interneurons while potentially maintaining motoneuron excitability. This may allow for adaptation of the locomotor pattern generated by interneuronal networks while helping to ensure the maintenance of overall motor output.

networks of neurons within the brain stem and spinal cord, called central pattern generators (CPGs), are responsible for the control of rhythmic motor behaviors such as locomotion and respiration. Neuromodulation is a process that allows these networks to adapt to the biomechanical demands of a range of motor tasks, and a wide range of neuromodulators has been identified to date (Miles and Sillar 2011). Purines, particularly ATP and adenosine, are one group of neuromodulators that play an important role in motor control (Brown and Dale 2000; Dale and Gilday 1996; Lorier et al. 2004).

The predominantly excitatory effects of ATP are mediated via P2 receptors throughout the CNS, while the generally inhibitory actions of adenosine result from binding to a family of P1 receptors (A_1_, A_2A_, A_2B_, and A_3_; Burnstock et al. 2011). Although ATP dominates purinergic signaling in many areas of the nervous system, there are also examples where adenosine acts as an important neuromodulator. For example, adenosine, rather than ATP, appears to dominate purinergic modulation in the hippocampus (Pascual et al. 2005; Zhang et al. 2003), retina (Newman 2003), calyx of Held (Wong et al. 2006), and cortex (Fellin et al. 2009). At the behavioral level, adenosine is known to play a role in the control of sleep (Basheer et al. 2004; Bjorness et al. 2009; Halassa et al. 2009; Nadjar et al. 2013; Porkka-Heiskanen et al. 1997; Thakkar et al. 2003), in relaying information regarding stress and pain (Bagley et al. 1999), and in the regulation of motor control systems (Brown and Dale 2000; Dale and Gilday 1996; Huxtable et al. 2009). Adenosine can be directly released from astrocytes, but the primary source of extracellular adenosine is thought to be ATP release from glia before breakdown to adenosine via ectonucleotidases (Hamilton and Attwell 2010).

Adenosine-mediated modulation has been well studied in the brain stem circuitry controlling mammalian respiration. Within these motor control circuits, glial-derived adenosine produces tonic depressive effects in rodents that are strongest at fetal stages (Herlenius and Lagercrantz 1999; Huxtable et al. 2010; Kawai et al. 1995; Lorier et al. 2007; Mironov et al. 1999; Schmidt et al. 1995). In addition to modulating respiratory frequency, presumably via actions on rhythm-generating neurons of the medullary pre-Bötzinger complex, adenosine also modulates the intensity of respiration-related output generated by motoneurons (Funk et al. 1997; Miles et al. 2002). Several cellular mechanisms are likely to underlie the depressive effects of adenosine on respiratory output including suppression of excitatory glutamatergic inputs to motoneurons (Bellingham and Berger 1994) and modulation of L-type Ca^2+^ channels and ATP-sensitive K^+^ channels (Mironov et al. 1999).

Within the spinal cord, adenosine is also important for the modulation of locomotor control networks. Of the four types of adenosine receptors, A_1_ and A_2_ receptors are most common in the spinal cord and are located diffusely throughout the ventral horn (Burnstock et al. 2011; Choca et al. 1987; Geiger et al. 1984). Behavioral effects of adenosine have been observed during swimming in frog tadpoles, where ATP first facilitates swimming by reducing voltage-activated K^+^ currents and increasing the excitability of neurons within the locomotor CPG. Ectonucleotidases then facilitate the breakdown of ATP to adenosine, which activates A_1_-type receptors, reducing voltage-activated Ca^2+^ currents and CPG excitability, resulting in the cessation of locomotor activity (Brown and Dale 2000, 2002; Dale and Gilday 1996).

We have recently shown that adenosine also modulates the frequency of motor output generated by the locomotor CPG of mice, most likely via the modulation of inhibitory components of the network (Acton and Miles 2015; Witts et al. 2012). We have demonstrated that glial-derived adenosine reduces or, perhaps in a physiological context, limits the frequency of locomotor-related output during ongoing network activity. However, the cellular mechanisms that underlie this spinal adenosine-mediated modulatory system remain unknown. In the present study we therefore utilized whole cell patch-clamp recordings of spinal motoneurons and ventral horn interneurons to investigate the consequences of adenosine receptor activation on the cellular components of spinal motor circuitry. We show that adenosine reduces the probability of transmitter release from presynaptic terminals and also hyperpolarizes interneurons. In contrast, adenosine depolarizes motoneurons and has no effect on the probability of transmitter release from last-order premotor interneurons. Thus we propose that a general inhibitory effect of adenosine on higher-order ventral horn interneurons leads to a reduced frequency of locomotor network output, while the simultaneous depolarization of motoneurons may help to ensure the maintenance of motor output and therefore an appropriate intensity of muscle activation.

## METHODS

### 

#### In vitro spinal cord slice preparation.

All methods required to obtain tissue for in vitro experiments were conducted in accordance with the UK Animals (Scientific Procedures) Act 1986 and were reviewed and approved by the University of St Andrews Animal Welfare and Ethics Committee. Spinal cord preparations were obtained from postnatal day (P)1–9 C57BL/6 mice with techniques similar to those described previously (Jiang et al. 1999). Briefly, animals were killed via cervical dislocation, decapitated, and eviscerated before spinal cords were isolated from the midcervical to upper sacral segments in a chamber containing dissecting artificial cerebrospinal fluid (aCSF; equilibrated with 95% O_2_-5% CO_2_, ∼4°C). Dorsal and ventral roots were trimmed, and the tissue was laid in 1% agar. A vibrating microtome (Leica VT1200) was used to obtain 300-μm transverse slices of the lumbar spinal cord. Slices were then transferred to recovery solution (equilibrated with 95% O_2_-5% CO_2_, ∼34°C) for 30 min to 1 h, before being secured in a recording chamber containing recording aCSF (equilibrated with 95% O_2_-5% CO_2_, room temperature).

#### Whole cell patch-clamp recordings.

Whole cell patch-clamp recordings were obtained from motoneurons and ventral horn interneurons visualized under infrared-differential interference contrast microscopy. We recorded from a heterogeneous population of interneurons throughout the ventral horn (average whole cell capacitance 32.9 ± 1.7 pF; average input resistance 366.8 ± 34.3 MΩ; *n* = 119). Interneurons were not readily classifiable into distinct subpopulations based on location, passive properties, or their responses to adenosine. Patch-clamp electrodes (3–5 MΩ) were pulled on a horizontal puller (Sutter Instrument, Novato, CA) from borosilicate glass (World Precision Instruments, Sarasota, FL). Signals were amplified and filtered (4-kHz low-pass Bessel filter) with a MultiClamp 700B amplifier (Molecular Devices) and acquired at ≥10 kHz with a Digidata 1440A A/D board and pCLAMP software (Molecular Devices). Details of voltage- and current-clamp protocols appear in results. We did not correct for the liquid junction potential, which was calculated as 14.2 mV for our solutions (Clampex JPCalcW).

#### Data analysis.

Whole cell patch-clamp recordings were analyzed with either Clampfit software (Molecular Devices) or, for analyses of synaptic events and miniature postsynaptic currents (mPSCs), the Mini Analysis Program (Synaptosoft, Fort Lee, NJ). One minute of recording from control, drug, and wash conditions was used for analysis of synaptic events, and 5 min of recording from control, drug, and wash conditions was used for analysis of mPSCs. The threshold for detection of events was set at three times the noise level for synaptic events and two times the noise level for mPSCs. Data are reported as means ± SE. Differences in means between control and drug were compared by Student's *t*-test. ANOVAs were used to compare means where there was more than one drug condition. The Kolmogorov-Smirnov test was used to test for differences in mPSC amplitude or interevent interval. Values of *P* < 0.05 were considered significant.

#### Solutions and drugs.

The aCSF solution used for dissecting contained (mM) 25 NaCl, 188 sucrose, 1.9 KCl, 1.2 NaH_2_PO_4_, 10 MgSO_4_, 1 CaCl_2_, 26 NaHCO_3_, 25 d-glucose, and 1.5 kynurenic acid (equilibrated with 95% O_2_-5% CO_2_). The aCSF solution used for recovery contained (in mM) 119 NaCl, 1.9 KCl, 1.2 NaH_2_PO_4_, 10 MgSO_4_, 1 CaCl_2_, 26 NaHCO_3_, 20 d-glucose, and 1.5 kynurenic acid, with 3% dextran added on the day of use (equilibrated with 95% O_2_-5% CO_2_). The aCSF solution used for recording contained (in mM) 127 NaCl, 3 KCl, 1.3 NaH_2_PO_4_, 1 MgCl_2_, 2 CaCl_2_, 26 NaHCO_3_, and 10 d-glucose (equilibrated with 95% O_2_-5% CO_2_). The standard patch-clamp pipette solution contained (in mM) 140 potassium methanesulfonate, 10 NaCl, 1 CaCl_2_, 10 HEPES, 1 EGTA, 3 Mg-ATP, and 0.4 GTP-Na_2_ (pH 7.2–7.3, adjusted with KOH). Adenosine, strychnine, picrotoxin, SCH58261, and 8-cyclopentyl-1,3-dipropylxanthine (DPCPX) were purchased from Sigma-Aldrich (St. Louis, MO); tetrodotoxin (TTX) was purchased from Tocris Bioscience (Bristol, UK). All drugs were made up fresh with aCSF, apart from strychnine and TTX, which were stored as frozen aliquots prior to their use, and DPCPX, which was made up fresh with DMSO. Unless otherwise stated, concentrations of drugs used were as follows: adenosine, 75 μM; DPCPX, 50 μM; SCH58261, 50 μM; TTX, 0.5 μM; strychnine, 1 μM; picrotoxin, 60 μM.

## RESULTS

### 

#### Adenosine hyperpolarizes spinal interneurons.

**Fig. 1.**
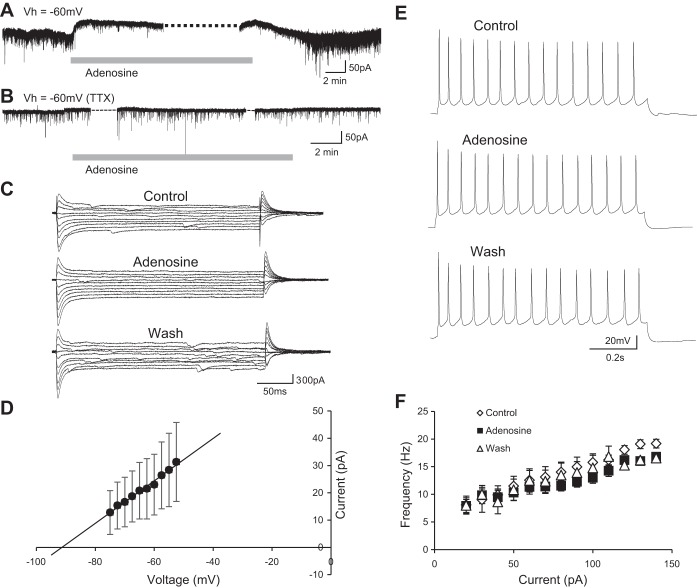
Adenosine hyperpolarizes interneurons by opening leak K^+^ channels. *A*: voltage-clamp recording from a ventral horn interneuron during a 15-min bath application of 75 μM adenosine [holding potential (*V*_h_) = −60 mV]. Adenosine induced an outward current in ventral horn interneurons held at −60 mV. *B*: voltage-clamp recording from a ventral horn interneuron during an application of 75 μM adenosine in the presence of TTX (0.5 μM; *V*_h_ = −60 mV). Adenosine no longer induced an outward current in the presence of TTX. *C*: example trace of 2.5-mV steps between −75 and −52.5 mV in control, adenosine, and wash conditions. Adenosine caused a reversible reduction in input resistance. *D*: averaged data (*n* = 22) showing the current-voltage (*I-V*) relationship of the adenosine-induced current revealed by subtracting control *I-V* relationships from those in the presence of adenosine. *E*: current-clamp recording showing repetitive action potential firing in a ventral horn interneuron in response to the injection of a square current pulse (110 pA) in control, adenosine, and wash. *F*: averaged data (*n* = 8) showing that adenosine had no effect on frequency-current relationships in control, adenosine, and wash (10-pA current steps from 20 to 140 pA).

To investigate the potential modulatory effects of adenosine receptor activation on the properties of individual spinal interneurons, we bath applied adenosine (75 μM; Witts et al. 2012) to spinal cord slice preparations while performing whole cell patch-clamp recordings of ventral horn interneurons. Despite the heterogeneity of the population of interneurons, responses to adenosine were similar across interneuron recordings. We first investigated whether adenosine receptor activation has any subthreshold effects on interneurons that might, for example, modulate their resting membrane potential. Voltage-clamp recordings of ventral horn interneurons held at −60 mV revealed that bath application of adenosine induced an outward current (43.2 ± 7.7 pA; *n* = 20 cells; Fig. 1*A*). This outward current was accompanied by a reduction in input resistance (control 469.3 ± 56.2 MΩ, adenosine 118.2 ± 72.0 MΩ), as calculated from current-voltage (*I-V*) relationships generated with a range of subthreshold voltage steps (2.5-mV steps from −75 to −52.5 mV; Fig. 1*C*). *I-V* relationships in control conditions were subtracted from those obtained in the presence of adenosine in order to isolate the adenosine-induced current (Fig. 1*D*). The *I-V* relationship of the adenosine-induced current revealed a reversal potential (−91 mV; Fig. 1*D*) near the equilibrium potential for K^+^ as calculated for our solutions with the Nernst equation (−98 mV). Thus our data support the concept that adenosine receptor activation leads to membrane hyperpolarization of ventral horn interneurons due to the opening of leak K^+^ channels. We next applied adenosine during the blockade of synaptic transmission to determine whether this outward current reflected direct, postsynaptic actions of adenosine on interneurons. Interestingly, the adenosine-induced current was blocked by TTX (0.5 μM, *n* = 6 cells; Fig. 1*B*).

We next utilized current-clamp mode to investigate the effects of adenosine receptor activation on interneuron input-output relationships. A series of square current pulses (20–140 pA; 10-pA increments, 1-s duration) were injected to induce firing in control conditions and during the bath application of adenosine (Fig. 1*D*). Adenosine had no significant effect on average rheobase current (control 41.3 ± 2.5 pA, adenosine 41.3 ± 2.5 pA; *n* = 8 interneurons) or the slope of frequency-current relationships of ventral horn interneurons (control 0.095 Hz/pA,; adenosine 0.070 Hz/pA; Fig. 1*F*; *n* = 8 interneurons). Therefore, although adenosine receptor activation hyperpolarizes ventral horn interneurons by mechanisms dependent on synaptic transmission, it does not lead to obvious changes in their input-output relationships as determined by stimulation using square current pulses.

#### Adenosine suppresses synaptic inputs to interneurons.

**Fig. 2.**
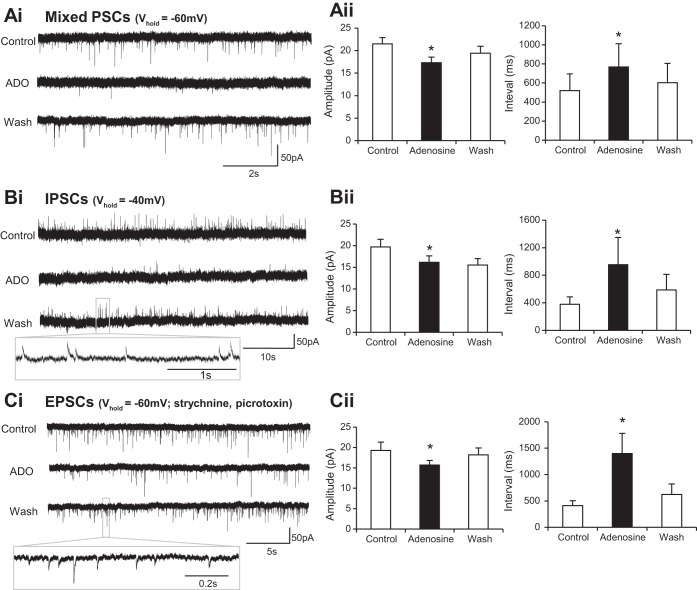
Adenosine reduces the frequency and amplitude of both excitatory and inhibitory inputs to interneurons. *Ai*: voltage-clamp recordings from an interneuron held at holding potential (*V*_hold_) of −60 mV revealing spontaneous synaptic inputs in control, adenosine (ADO), and wash conditions. *Aii*: averaged data showing that application of adenosine caused a reduction in the amplitude of synaptic events and an increase in the interval between events. Note: these synaptic events represent a mixture of excitatory (EPSCs) and inhibitory (IPSCs) postsynaptic currents (both are depolarizing at this potential in our recording solutions). *Bi*: voltage-clamp recordings taken from ventral horn interneurons held at −40 mV, a potential at which IPSCs are hyperpolarizing and therefore distinguishable from EPSCs. *Bii*: averaged data showing that application of adenosine led to an irreversible reduction in IPSC amplitude and a reversible increase in interval between IPSCs. *Ci*: voltage-clamp recordings of EPSCs taken at −60 mV while inhibitory transmission was blocked with 1 μM strychnine (glycine receptor antagonist) and 60 μM picrotoxin (GABA receptor antagonist). *Cii*: averaged data showing that application of adenosine led to a reversible reduction in EPSC amplitude and a reversible increase in interval between EPSCs. *Significantly different from control.

Given the evidence of widespread involvement of adenosine receptor activation in the modulation of synaptic activity (reviewed by Burnstock et al. 2011), we next investigated whether adenosine affects synaptic inputs to ventral horn interneurons. Voltage-clamp recordings from interneurons held at −60 mV revealed an adenosine-induced decrease in the amplitude of synaptic events (control 21.5 ± 1.4 pA, adenosine 17.3 ± 1.2 pA; *n* = 20 cells) along with an increase in interevent interval (control 519.1 ± 176.0 ms, adenosine 767.9 ± 244.2 ms; *n* = 20 cells; Fig. 2, *Ai* and *Aii*). Although these data clearly demonstrate modulation of synaptic activity, they do not allow separation of the effects on inhibitory and excitatory inputs because due to the reversal potential of Cl^−^ in our recording solutions both types of inputs are likely to be depolarizing at a holding potential of −60 mV. We therefore went on to dissect out the effects of adenosine receptor activation on excitatory (EPSCs) and inhibitory (IPSCs) postsynaptic currents. IPSCs were isolated by using a holding potential of −40 mV, at which IPSCs are hyperpolarizing and therefore distinguishable from EPSCs. We found that adenosine caused a reduction in the amplitude of IPSCs (control 19.7 ± 1.8 pA, adenosine 16.2 ± 1.5 pA; *n* = 10 cells) and an increase in the interval between IPSCs (control 379.3 ± 107.2 ms, adenosine 952.5 ± 397.1 ms; *n* = 10 cells; Fig. 2, *Bi* and *Bii*). EPSCs were then isolated by performing voltage-clamp recordings at −60 mV while blocking inhibitory transmission with 1 μM strychnine (glycine receptor antagonist) and 60 μM picrotoxin (GABA receptor antagonist). Under these conditions application of adenosine caused a reduction in EPSC amplitude (control 19.3 ± 2.1 pA, adenosine 15.7 ± 1.1 pA; *n* = 10 cells) and increased the interval between excitatory inputs (control 412.0 ± 92.1 ms, adenosine 1,398.8 ± 381.7 ms; *n* = 10 cells; Fig. 2, *Ci* and *Cii*). In addition, inward currents were still observed when adenosine was applied in the absence of inhibitory transmission (30.8 ± 1.3 pA; *n* = 10 cells; data not shown). Taken together, these data demonstrate that adenosine receptor activation has a general suppressive effect on synaptic input to ventral horn interneurons.

**Fig. 3.**
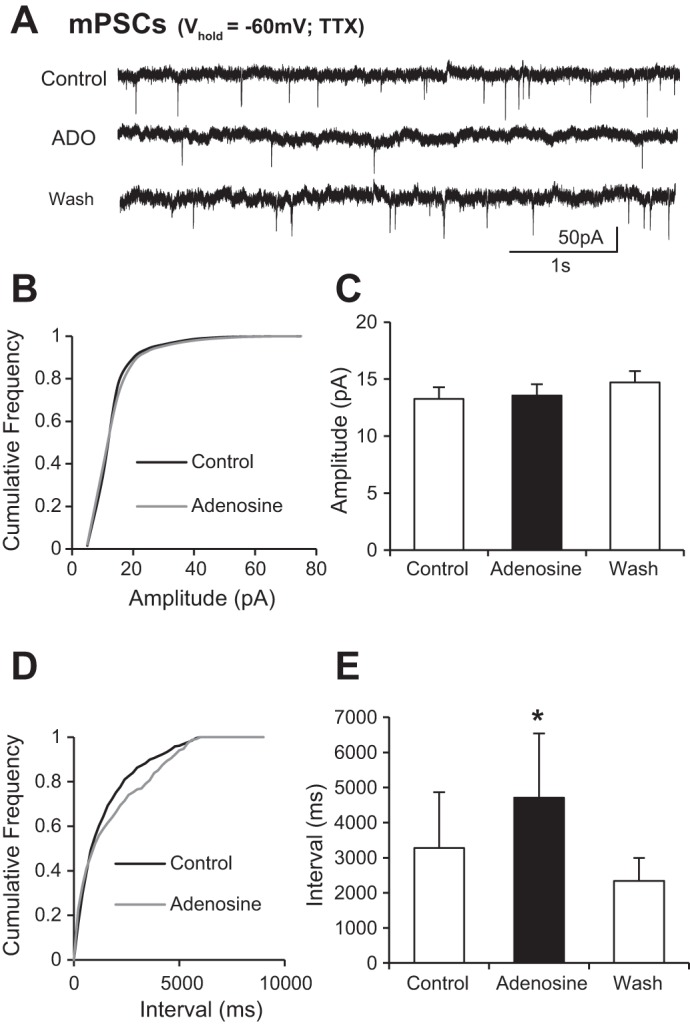
Adenosine reduces the frequency of miniature PSCs (mPSCs) recorded from interneurons. *A*: voltage-clamp recordings from an interneuron held at −60 mV in the presence of TTX, where sodium currents are blocked and so action potential-independent, mPSCs are isolated. *B*: averaged cumulative probability plot showing no change in amplitude of miniature postsynaptic potentials (*n* = 8 cells). *C*: averaged data showing that application of adenosine has no effect on the amplitude of mPSCs. *D*: averaged cumulative probability plot showing an increase in the interval between events in the presence of adenosine. *E*: averaged data showing that application of adenosine causes an increase in the interval between mPSCs. These data support involvement of presynaptic adenosine receptors in adenosine-mediated modulation of synaptic transmission. *Significantly different from control.

Reductions in synaptic activity could reflect diminished output from presynaptic neurons due to adenosine-induced hyperpolarization or direct modulation of synaptic transmission via pre- and/or postsynaptic mechanisms. To distinguish between these possibilities we next investigated the effects of adenosine application on action potential-independent mPSCs recorded from ventral horn interneurons in the presence of TTX (0.5 μM). Cumulative frequency plots and average values measured in each condition were used to assess the effects of adenosine on mPSC amplitude and frequency (Fig. 3). Adenosine was found to have no effect on mPSC amplitude (Fig. 3, *B* and *C*; control 13.3 ± 1.7 pA, adenosine 13.6 ± 2.2 pA; *n* = 8 cells). In comparison, adenosine application significantly increased the interval between mPSCs (Fig. 3, *D* and *E*; control 3,278.1 ± 1,591.2 ms, adenosine 4,711.3 ± 1,824.1 ms; *n* = 8 cells). These data therefore support the concept that the primary effect of adenosine on synaptic transmission between spinal interneurons involves activation of presynaptic receptors that in turn reduce the probability of transmitter release.

#### Adenosine-mediated modulation of interneurons involves A_1_ receptors.

**Fig. 4.**
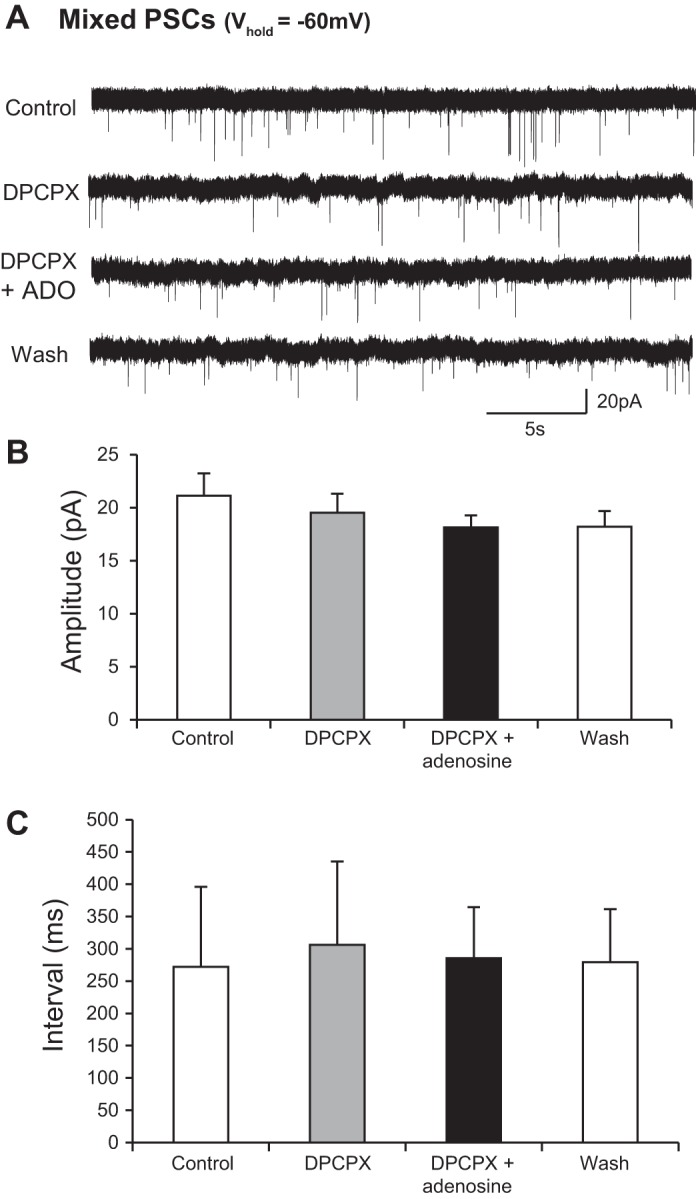
An A_1_ receptor antagonist blocks the effect of adenosine on synaptic inputs to interneurons. *A*: voltage-clamp recordings from an interneuron held at −60 mV revealing spontaneous synaptic inputs in control, with the A_1_ adenosine receptor antagonist DPCPX, in DPCPX and adenosine, and wash. *B*: averaged data showing that there was no change in the amplitude of synaptic inputs in the presence of DPCPX and that DPCPX blocked the previously observed effects of adenosine (*n* = 10). *C*: averaged data showing that there was no change in the interval between synaptic inputs in the presence of DPCPX and that DPCPX blocked the previously observed effects of adenosine (*n* = 10).

We next investigated which receptor subtypes are involved in the modulatory effects of adenosine on ventral horn interneurons. Given widespread expression of A_1_-type receptors in the rodent spinal cord (Deuchars et al. 2001) and our previous findings demonstrating A_1_ receptor-mediated modulation of locomotor network output (Witts et al. 2012), we first tested whether the A_1_ receptor antagonist DPCPX could block adenosine-mediated modulation. In the presence of DPCPX [100 nM (*n* = 11 interneurons) or 50 μM (*n* = 8 interneurons)], adenosine failed to induce an outward current and had no effect on mixed IPSCs and EPSCs recorded from interneurons held at −60 mV (Fig. 4). DPCPX alone had no effect on synaptic inputs, nor did it induce any subthreshold currents, indicating a lack of tonic activation of A_1_ receptors in our slice preparations. We next investigated whether adenosine acted exclusively via A_1_ receptors by applying adenosine in the presence of the adenosine A_2A_ receptor antagonist SCH58261 (50 μM). In the presence of SCH58261, adenosine induced an inward current (25.9 ± 1.4 pA; *n* = 5 cells). Adenosine also reduced the amplitude of PSCs (control 10.1 ± 2.0 pA, adenosine 8.9 ± 1.9 pA; *n* = 5 cells) and increased the average interval between PSCs (control 6,552.1 ± 1,571.9 ms, adenosine 8,940.2 ± 1,700.5 ms; *n* = 5 cells; data not shown). It therefore appears that the effects of adenosine in interneurons are predominantly mediated by A_1_ receptors.

#### Adenosine depolarizes motoneurons.

**Fig. 5.**
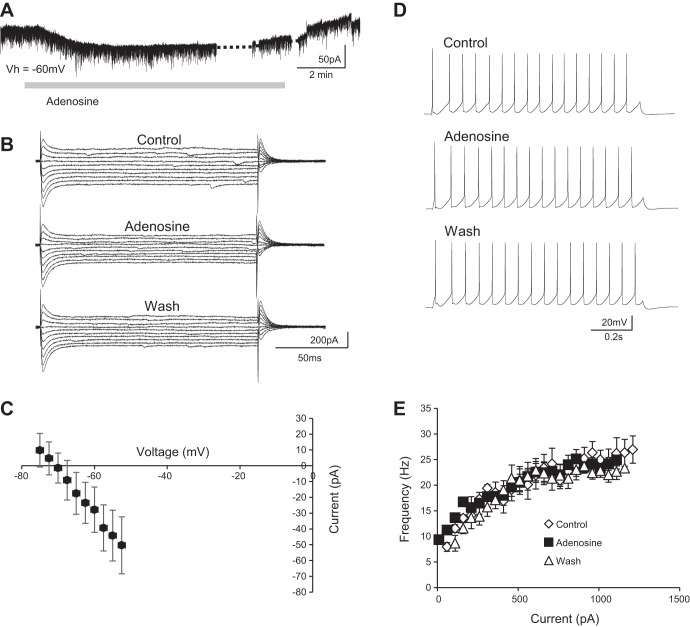
Adenosine depolarizes spinal motoneurons. *A*: voltage-clamp recording from a spinal motoneuron during bath application of 75 μM adenosine (*V*_h_ = −60 mV). Adenosine induced an inward current in motoneurons held at −60 mV. *B*: example trace of 2.5-mV steps between −75 and −52.5 mV in control, adenosine, and wash conditions. Adenosine caused an increase in input resistance. *C*: averaged data (*n* = 11) showing the *I-V* relationship of the adenosine-induced current revealed by subtracting control *I-V* relationships from those in the presence of adenosine. *D*: current-clamp recording showing repetitive action potential firing in a ventral horn interneuron in response to the injection of a square current pulse in control, adenosine, and wash. *E*: averaged data (*n* = 8) for control, adenosine, and wash showing that adenosine had no effect on frequency-current relationships.

Having observed the effects of adenosine on interneurons, we next assessed whether adenosine receptor activation also modulates the properties of motoneurons or the synaptic inputs they receive. Whole cell patch-clamp recordings of motoneurons were first utilized to investigate whether adenosine receptor activation induces subthreshold responses in motoneurons. In contrast to ventral horn interneurons, when motoneurons were held at −60 mV in voltage-clamp mode adenosine induced an inward current (47.6 ± 8.7 pA, *n* = 10; Fig. 5*A*). This inward current was accompanied by an increase in input resistance (control 90.6 ± 33.5 MΩ, adenosine 110.5 ± 27.2 MΩ), as revealed by analysis of *I-V* relationships generated from subthreshold voltage steps (2.5-mV increments, ranging from −75 to −52.5 mV; Fig. 5, *B* and *C*). *I-V* relationships obtained in control conditions were subtracted from those obtained during adenosine application in order to isolate the adenosine-induced current. The *I-V* relationship of the adenosine-induced current had a reversal potential of −71 mV (Fig. 5*C*). We also applied adenosine in the presence of TTX (0.5 μM) and found that adenosine no longer induced an inward current (data not shown). Given that this current appeared to be dependent on synaptic transmission and had a reversal potential near that calculated for Cl^−^ in our solutions (−62 mV), we hypothesized that it may reflect the blockade of a tonic inhibitory input to motoneurons. However, adenosine applied in the presence of strychnine and picrotoxin still induced an inward current in motoneurons (42.4 ± 15.9 pA, *n* = 5; data not shown).

Next, motoneuron recordings were performed in current-clamp mode to investigate the effects of adenosine receptor activation on motoneuron input-output relationships. Square current pulses (ranging from 10 to 1,210 pA; 50-pA increments, 1-s duration) were injected to induce repetitive firing in control conditions and during the bath application of adenosine (Fig. 5*D*). Adenosine had no significant effect on rheobase current (control 353.8 ± 34.5 pA, adenosine 353.8 ± 36.3 pA; *n* = 8 motoneurons) or the slope of frequency-current relationships of ventral horn interneurons (control 0.013 Hz/pA, adenosine 0.012 Hz/pA; Fig. 5, *D* and *E*; *n* = 8 motoneurons). Therefore, although adenosine receptor activation depolarizes motoneurons, it does not lead to obvious changes in their input-output relationships as determined by stimulation using square current pulses.

#### Adenosine modulates synaptic input but not mPSCs recorded from motoneurons.

**Fig. 6.**
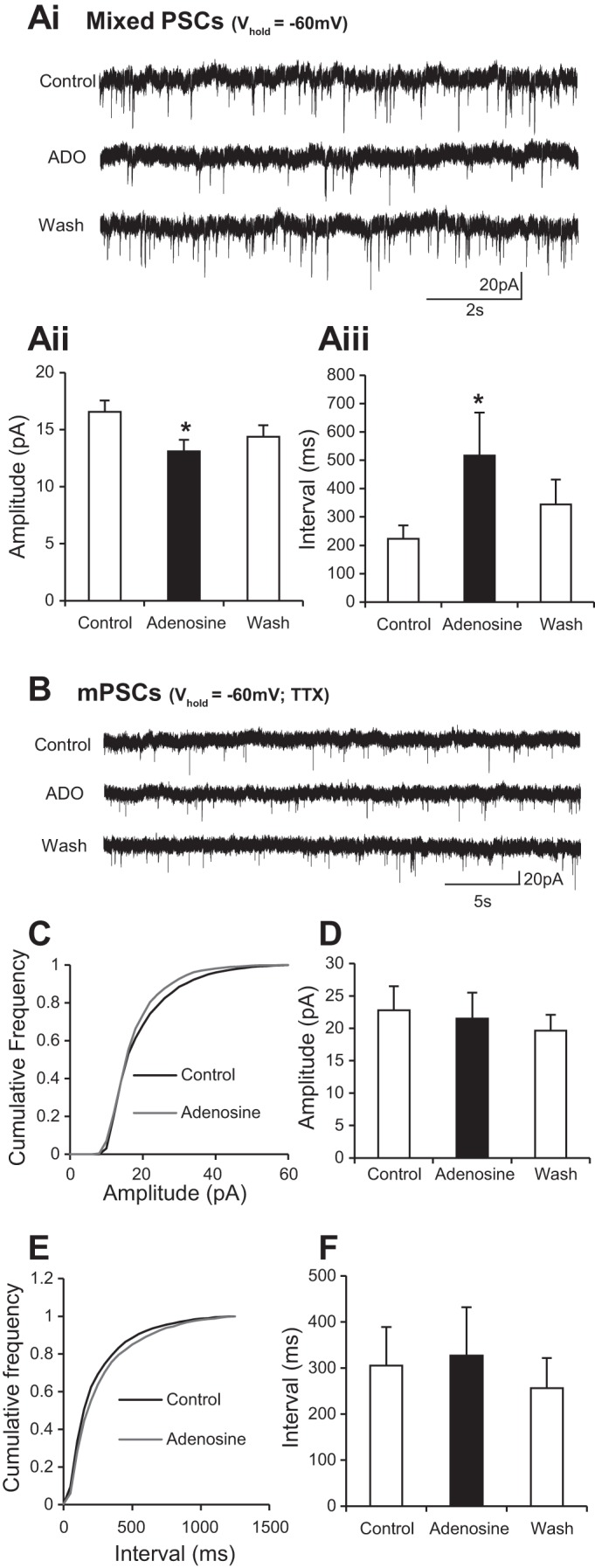
Adenosine reduces the frequency and amplitude of synaptic inputs to motoneurons but does not affect mPSCs. *Ai*: voltage-clamp recordings from a motoneuron held at −60 mV revealing spontaneous synaptic inputs in control, adenosine, and wash conditions. *Aii*: averaged data showing that application of adenosine caused a reduction in the amplitude of synaptic events (*n* = 12 cells). *Aiii*: averaged data showing that application of adenosine caused an increase in the interval between events (*n* = 12). *B*: voltage-clamp recordings of mPSCs in a motoneuron held at −60 mV in the presence of TTX. *C*: averaged cumulative probability plot showing no change in amplitude of miniature postsynaptic potentials. *D*: averaged data showing that application of adenosine does not affect the amplitude of mPSCs. *E*: averaged cumulative probability plot showing no change in the interval between events. *F*: averaged data showing that application of adenosine does not affect the interval between mPSCs. These data suggest that adenosine does not directly affect synaptic transmission onto motoneurons. *Significantly different from control.

Given the effects of adenosine receptor activation on synaptic activity recorded from interneurons, we also assessed whether adenosine modulates synaptic inputs received by motoneurons. Voltage-clamp recordings of motoneurons held at −60 mV once again demonstrated suppressive effects of adenosine on synaptic input with a reduction in amplitude (control 16.6 ± 1.9 pA, adenosine 13.1 ± 1.0 pA; *n* = 10; Fig. 6, *Ai* and *Aii*) and an increase in interevent interval of synaptic inputs received by motoneurons (control 222.6 ± 47.9 ms, adenosine 516.6 ± 151.5 ms; *n* = 10; Fig. 6, *Ai* and *Aiii*). However, in contrast to analyses of interneurons, recordings of mPSCs from motoneurons held at −60 mV in the presence of TTX revealed that adenosine had no effect on the amplitude (Fig. 6, *B–D*) or interevent interval (Fig. 6, *B*, *E*, and *F*; *n* = 8) of mPSCs. These data suggest that adenosine-mediated reductions in synaptic activity recorded from motoneurons most likely involve reductions in the excitability and synaptic activity in premotor networks rather than direct modulation of last-order synapses on motoneurons.

#### Adenosine-mediated modulation of motoneurons involves A_1_ receptors.

**Fig. 7.**
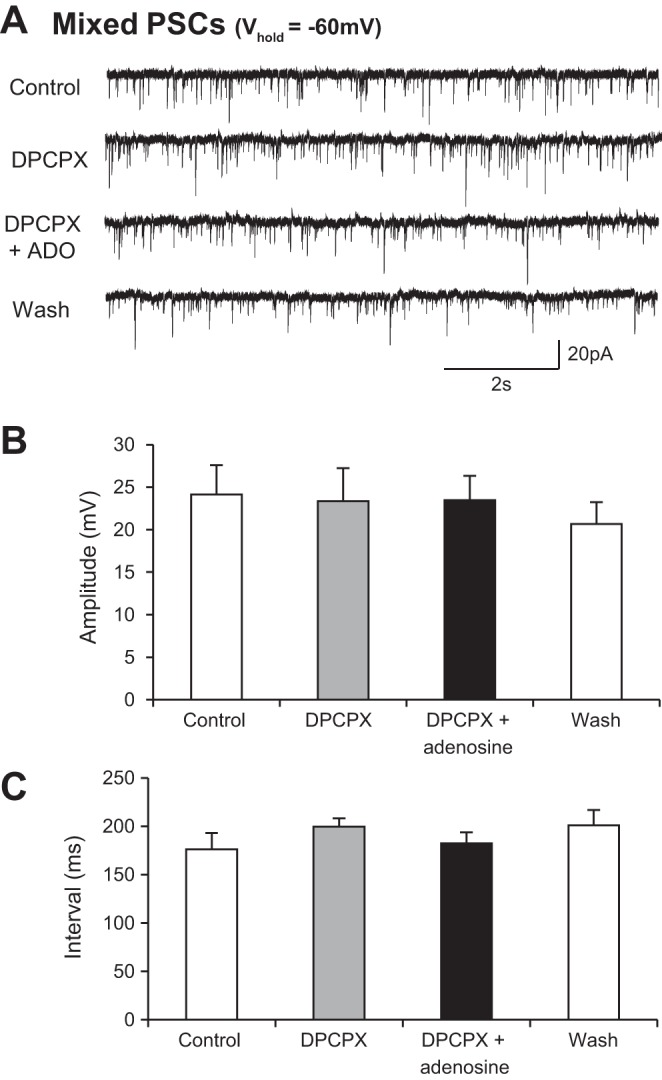
DPCPX blocks the effect of adenosine on synaptic inputs to motoneurons. *A*: voltage-clamp recordings from a motoneuron held at −60 mV revealing spontaneous synaptic inputs in control, DPCPX, DPCPX and adenosine, and wash conditions. *B*: averaged data showing that there was no change in the amplitude of synaptic inputs in the presence of DPCPX and that DPCPX blocked the previously observed effects of adenosine (*n* = 8). *C*: averaged data showing that there was no change in the interval between synaptic inputs in the presence of DPCPX and that DPCPX blocked the previously observed effects of adenosine (*n* = 8).

Finally, we again assessed whether adenosine-mediated modulation of motoneurons and the inputs they receive reflects activation of A_1_ receptors. In the presence of the A_1_ receptor antagonist DPCPX [100 nM (*n* = 7 motoneurons) or 50 μM (*n* = 8 motoneurons)], adenosine no longer induced an inward current in motoneurons and synaptic activity was unchanged (Fig. 7). DPCPX again had no effect on its own, indicating no endogenous activation of A_1_ receptors in our slice preparations. We next investigated whether adenosine acted exclusively via A_1_ receptors by applying adenosine in the presence of the adenosine A_2A_ receptor antagonist SCH58261 (50 μM). In the presence of SCH58261, adenosine still induced an inward current (22.7 ± 3.9 pA; *n* = 5 cells), reduced the amplitude of PSCs (control 11.5 ± 1.1 pA, adenosine 8.9 ± 0.9 pA; *n* = 7 cells), and increased the average interval between PSCs (control 3,412.7 ± 414.8 ms, adenosine 9,540.1 ± 1,048.8 ms; *n* = 7 cells). These data suggest that the effects of adenosine observed in motoneurons are predominantly mediated by A_1_ receptors.

## DISCUSSION

Adenosine has been shown to modulate spinal circuits responsible for the generation of tadpole swimming, mammalian respiration, and, most recently, walking in neonatal mice (Acton and Miles 2015; Dale and Gilday 1996; Lorier et al. 2007; Taccola et al. 2012; Witts et al. 2012). Here we report potential cellular mechanisms for purinergic modulation of mammalian locomotor control circuitry. We found that the effects of adenosine receptor activation differ between ventral horn interneurons and spinal motoneurons despite acting via A_1_ receptors in both cases. Adenosine hyperpolarizes ventral horn interneurons and modulates their synaptic transmission via actions on presynaptic A_1_ receptors. In contrast, A_1_ receptor activation depolarizes motoneurons but does not directly affect fast synaptic transmission between last-order interneurons and motoneurons. Interestingly, currents induced in interneurons and motoneurons were blocked by TTX, implying a role for synaptic transmission in these responses.

We have previously shown that bath application of adenosine to in vitro preparations of the whole spinal cord reduces the frequency of locomotor output generated by the spinal locomotor CPG (Witts et al. 2012). We proposed that this was caused by a general inhibition of CPG neurons. In the present study, we have shown that ventral horn interneurons are indeed inhibited by A_1_ adenosine receptor activation. Application of adenosine led to the hyperpolarization of interneurons and a general reduction in synaptic activity, likely due to presynaptic inhibition of transmitter release. Both of these effects were blocked by the A_1_ receptor antagonist DPCPX (100 nM). Although adenosine-induced currents remained in the presence of the A_2A_ receptor antagonist SCH58261, they were reduced in magnitude, suggesting that there may also be some involvement of A_2A_ receptors. However, given the complete block by 100 nM DPCPX, the reduction in current magnitude in the presence of SCH58261 (50 μM) may reflect nonspecific actions on A_1_ receptors. Our evidence of a primary role for A_1_ receptors in the modulation of ventral horn interneurons and locomotor output is consistent with reports of extensive expression of A_1_ receptors throughout the rodent spinal cord (Deuchars et al. 2001) and A_1_-mediated modulation of rat spinal neurons (Miyazaki et al. 2008). Furthermore, our findings in the mouse spinal cord and previous reports investigating tadpole swimming and rodent respiration (Dale and Gilday 1996; Lorier et al. 2007) are consistent with a general inhibitory role for A_1_ receptors in neuronal circuits throughout the CNS (Burnstock et al. 2011).

Adenosine has often been shown to have global modulatory effects on neuronal networks throughout the CNS (Burnstock et al. 2011). This likely explains why, despite the heterogeneous nature of ventral horn interneurons (see Arber 2012 for a recent review), the effects of adenosine receptor activation were consistent across the different interneurons, likely both inhibitory and excitatory, from which we recorded. Although a large undertaking, it would be interesting in future work to investigate the effects of adenosine on the wide range of specific interneuron populations defined with genetic markers (Kiehn 2011). This would elucidate whether adenosine truly has the same effects on all ventral horn interneurons or, if there is variation in responses, may provide insight into which interneurons are critical components of the locomotor CPG circuitry.

Despite the apparent homogeneity of responses to adenosine among ventral horn interneurons, we observed a very clear difference between the responses of motoneurons and interneurons. Motoneurons showed a clear, reversible depolarization, rather than a hyperpolarization, in response to bath application of adenosine. Although previous work supports a postsynaptic A_1_ receptor-mediated modulation of K^+^ currents in rat spinal neurons (Miyazaki et al. 2008), we found that currents induced in interneurons and motoneurons were blocked by TTX, implying a role for synaptic transmission in these responses. Although perhaps the simplest explanation in motoneurons was that adenosine reduced tonic inhibitory inputs, the finding that adenosine-mediated currents persisted in the presence of strychnine and picrotoxin did not support this mechanism. Although it is not possible to determine the exact mechanisms from our experiments, one possible explanation is that in both motoneurons and interneurons adenosine acts as a metamodulator, modulating the effects of other neuromodulatory inputs, which in turn regulate currents involved in setting the resting membrane potential. This phenomenon of metamodulation has already been reported in locomotor networks of the tadpole spinal cord, where nitric oxide modulates the actions of norepinephrine (McLean and Sillar 2004). In addition, adenosine has been shown to act as a metamodulator in other systems (Ribeiro and Sebastiao 2010).

The differential effect of adenosine on motoneurons and interneurons may relate to the importance of maintaining motor output and hence muscle contraction during locomotion. Adenosine-mediated depolarization along with associated increases in input resistance may help to ensure that motoneurons remain responsive to synaptic inputs despite a generalized inhibition of other components of spinal motor circuitry. Although we did not observe obvious changes in the frequency-current relationships of motoneurons upon adenosine receptor activation, one would still expect motoneurons to be more excitable because of their depolarization and higher resistance. Our lack of detection of clear changes in rheobase or frequency-current relationships may reflect the limited resolution of graduated steps of current injection, particularly when dealing with relatively small depolarizing currents.

The dual, opposing effects of adenosine on interneurons and motoneurons that we have reported here provide further evidence that single neuromodulators can have multiple effects within distinct neuronal networks (Harris-Warrick 2011). Modulators can act differently according to the state of a neuronal circuit (e.g., Doi and Ramirez 2010), receptor subtype activated (e.g., Duarte-Araujo et al. 2004), or downstream pathway engaged (e.g., Iwagaki and Miles 2011; Nanou and El Manira 2010). Given the large number of modulators identified to date, neuromodulation therefore has the potential to facilitate the production of commands for a wide range of movements. Here we show differential effects of adenosine receptor activation, presumably due to the engagement of different pathways linking A_1_ receptors to spinal motoneurons vs. interneurons. Previous work has also shown that adenosine can have opposing actions within the rat myenteric plexus depending upon its source and the exact receptor type activated (Duarte-Araujo et al. 2004). Thus, like other single modulators, adenosine has the potential in its own right to facilitate the production of a variety of outputs from motor networks.

We have previously demonstrated that endogenous adenosine released from within isolated spinal cord preparations modulates locomotor circuitry (Witts et al. 2012). Furthermore, we provided evidence that this endogenous adenosine is derived from the breakdown of ATP following its release from glial cells. In the present study, application of the A_1_ receptor antagonist DPCPX alone had no effect on synaptic transmission or the intrinsic properties of interneurons or motoneurons, suggesting a lack of endogenous adenosine in our in vitro slice preparations. Thus it seems likely that whole network activity is needed to stimulate purine release from glia. Further support of a role for glial-derived adenosine in the mouse spinal cord has recently been provided by a study showing that stimulation of astrocytes leads to an adenosine-mediated inhibition of synaptic transmission between ventral horn neurons (Carlsen and Perrier 2014). In agreement with our present findings, this study also concluded that this inhibition involved presynaptic A_1_ receptors. It remains unclear, however, what physiological signals normally stimulate the release of gliotransmitters, such as purines, during network activity.

The release of adenosine has been associated with a range of pathological conditions including spinal cord injury (Burnstock et al. 2011; McAdoo et al. 2000). Subsequent activation of adenosine receptors is most often thought to be neuroprotective (Burnstock et al. 2011). However, controversy remains regarding the potential neuroprotective role of adenosine receptors in the spinal cord (Rivera-Oliver and Diaz-Rios 2014). Neuroprotective effects of A_1_ receptor activation in the brain are thought to involve concurrent presynaptic inhibition of transmitter release and hyperpolarization of postsynaptic neurons (Burnstock et al. 2011). Given that we have shown similar effects of A_1_ receptor activation on ventral horn interneurons, our data support A_1_ receptors as a potential target for the treatment of pathological conditions affecting the spinal cord. This might include neurodegenerative diseases such as amyotrophic lateral sclerosis (ALS), since recent work has shown that blockade of adenosine receptors shortens survival in ALS model mice (Potenza et al. 2013). Further analysis of the effects of purinergic signaling in the mammalian spinal cord is therefore likely to be important not only for advancing our understanding of the neural control of movement but also for the treatment of injury and disease affecting the spinal cord.

## GRANTS

We are grateful for support from the Wellcome Trust.

## DISCLOSURES

No conflicts of interest, financial or otherwise, are declared by the author(s).

## AUTHOR CONTRIBUTIONS

Author contributions: E.C.W., F.N., and G.B.M. conception and design of research; E.C.W. and F.N. performed experiments; E.C.W. and F.N. analyzed data; E.C.W., F.N., and G.B.M. interpreted results of experiments; E.C.W. and G.B.M. prepared figures; E.C.W. and G.B.M. drafted manuscript; E.C.W., F.N., and G.B.M. edited and revised manuscript; E.C.W., F.N., and G.B.M. approved final version of manuscript.
